# Induction of interleukin-6 by SPZ1-mediated Wnt5a signaling boosts progression of nasopharyngeal carcinoma cells

**DOI:** 10.7150/jca.99648

**Published:** 2024-10-07

**Authors:** Xiaoxia Zeng, Dunhui Yang, Kang Li, Jin Zhang, Dayang Qin, Zhen Wang, Fang Ma, Xianqin Liao, Xiao-Yu Liu, Xianhai Zeng, Peng Zhang

**Affiliations:** 1 Department of Otolaryngology, Longgang Otolaryngology hospital & Shenzhen Key Laboratory of Otolaryngology, Shenzhen Institute of Otolaryngology, Shenzhen, Guangdong, China.; 2 Department of Otolaryngology, The Second People's Hospital of Yibin, Yibin, Sichuan, China.; 3 Department of Otolaryngology, the First People's Hospital of Qinzhou, the Tenth Affiliated Hospital of Guangxi Medical University, Qinzhou, Guangxi, China.; 4 School of Medicine, Southern University of Science and Technology and Shenzhen Middle School, Shenzhen, Guangdong, China.

**Keywords:** nasopharyngeal carcinoma, SPZ1, Wnt5a, interleukin-6, tumorigenesis

## Abstract

Nasopharyngeal carcinoma (NPC) is a common malignancy in Southeast Asia, and in the Guangxi and Guangdong provinces of China. The spermatogenic transcription factor zip 1 (SPZ1) is a member of bHLH zip family, and promotes tumorigenesis in the liver, colon and breast tissues. However, the role of SPZ1 in the progression of NPC is unclear. In this study, we found that SPZ1 mRNA and protein levels were significantly upregulated in NPC tissues compared to the normal nasopharyngeal tissues. Furthermore, SPZ1 knockdown in NPC cell lines inhibited proliferation, epithelial-mesenchymal transition, migration, and invasion *in vitro*, and suppressed tumorigenesis in an *in vivo* model. On the other hand, SPZ1 overexpression facilitated the growth of NPC cells. Mechanistically, SPZ1-driven progression of NPC is dependent on the Wnt5a/interleukin-6 (IL-6) signaling pathway. Consistent with this, IL-6 levels were significantly increased in NPC tissues and correlated positively with SPZ1 expression. Taken together, our findings suggest that SPZ1 mediates NPC progression through Wnt5a/IL-6 signaling, and the SPZ1/Wnt5a/IL-6 axis is a potential therapeutic target for NPC.

## Introduction

Nasopharyngeal carcinoma (NPC) is a common malignancy that originates from the epithelial cells of the nasopharynx 1. It is endemic in some regions, including the Guangxi and Guangdong provinces in China 2,3. Epstein-Barr virus infection, presence of susceptibility genes, environmental factors and diet are associated with NPC progression 4. Radio/chemotherapy is the standard treatment for locoregionally advanced NPC. However, most NPC patients are diagnosed in the late stage of the disease, which is characterized by aggressive proliferation and metastasis of the tumor cells, and resistance to radio/chemotherapy. In fact, treatment resistance accounts for most NPC-related deaths 5. Despite efforts to discover novel therapeutic targets, the pathogenesis of NPC is complex. Therefore, it is critical to identify the mechanisms underlying progression of NPC in order to develop targeted therapies.

The basic helix-loop-helix (bHLH) zip proteins regulate various physiological and pathological processes 6, including cell proliferation, trans-differentiation, and tumorigenesis 7. The spermatogenic transcription factor zip 1 (SPZ1) is a member of the bHLH zip family, and is predominantly expressed in the testis 8. The SPZ1 transgenic mice are highly susceptible to developing tumors 9,10. In addition, Wang *et al.* found that SPZ1 controls human liver oncogenesis by regulating TWIST-mediated epithelial-mesenchymal transition (EMT) 11. The same group also demonstrated that activation of the SPZ1-TWIST complex by TIP60-dependent acetylation promotes EMT and metastasis of liver cancer cells 12. In our previous studies, we showed that SPZ1 contributes to the invasion and chemoresistance of breast cancer cells, and protects colorectal cancer cells against apoptosis 13,14. However, the potential role of SPZ1 in NPC progression, and the underlying molecular mechanisms, remain unclear.

In this study, we found that SPZ1 is highly expressed in NPC tissues, and promotes the proliferation, EMT, migration and invasion of NPC cells via the Wnt5a/ IL-6 signaling pathway. This study provides new insights into the role of SPZ1 in the progression of NPC, and its potential as a therapeutic target.

## Materials and methods

### Ethics statement and patients

Experiments involving tissue slides were approved by the Review Board of Longgang Otolaryngology hospital, and the experiments conformed to the principles contained in the World Medical Association Declaration of Helsinki. Informed consent was requested and obtained from all human participants for collecting anonymous specimens.

### Cell culture

The human NPC cell lines 5-8F, 6-10B and C666-1 were obtained from BeNa Culture Collection, Jiangsu, China. CNE2 and HONE1 were obtained from College of Pharmacy, Guilin Medical University, Guangxi, China. CNE1 was obtainedfrom School of Basic Medical Sciences, Guangzhou University of Chinese Medicine, Guangzhou, China^15^. The cells were cultured in DMEM or RPMI 1640 medium supplemented with 10% FBS (Gibco) at 37°C in a humidified incubator (Thermo Fisher Scientific) with 5% CO_2_.

### Real-time PCR

Total RNA was extracted from the cultured cells using RNeasy Mini Kit (Qiagen) and reverse transcription was performed with RT Master Mix for qPCR kit (MedChemExpress). Real-time PCR was performed using SYBR Green qPCR Master Mix (MedChemExpress) on the ABI7500 FAST Real Time PCR system (Applied Biosystems)^15^. Primer sequences were listed in the supplemental Table S1.

### Western blotting

The cultured cells were homogenized in RIPA lysis buffer (Beyotime Biotech) to extract the protein fraction. Equal amounts of protein per sample were separated by 10% SDS-PAGE, and then transferred onto PVDF membrane (Millipore). After blocking with 5% BSA in TBST for 1h at room temperature, the membranes were incubated overnight with anti-SPZ1 (1:1000, GeneTex) and anti-ACTB (1:3000, Santa Cruz) antibodies at 4°C. Following incubation with HRP-conjugated secondary antibodies (Cell Signaling) at room temperature for 2h, the protein bands were detected using Pierce™ ECL Western Blotting Substrate (Thermo Scientific).

### siRNA transfection

The cells were transfected with gene-specific or scrambled siRNAs using Lipofectamine™ RNAiMAX (Invitrogen) according to the manufacturer's instructions 14. The siRNAs were synthesized by Shanghai GenePharma Co. Ltd. Target sequences were listed in the supplemental Table S2. The cells were harvested 48 or 72h after transfection, and total RNA or protein was isolated as described in the previous sections.

### Overexpression of SPZ1

Cells were transfected with the plasmid carrying full-length human SPZ1 cDNA (GenBank NM 032567.2) or empty vector (OriGene Technologies Inc) using Lipofectamine™ 2000 reagent (Invitrogen) according to the manufacturer's instructions. The stably transfected cells were screened with 800μg/ml G418 sulfate (Sigma) 14.

### Migration and invasion assays

Cell migration was determined using transwell chambers (Corning) as described previously 13. Briefly, the cells were seeded into the upper chambers in serum-free medium, and the lower chambers were filled with complete medium (with 10% FBS). After incubating for 24 h, the cells remaining in the upper chamber were removed with a cotton swab, and the cells that migrated through the membrane were fixed with 4% paraformaldehyde and stained with crystal violet. For the cell invasion assay, the upper chambers were coated with Matrigel (BD Biosciences) prior to seeding the cells. The remainder of the procedure was same as that of the migration assay.

### Enzyme-linked immunosorbent assay (ELISA)

Wnt5a and IL-6 levels were estimated using specific ELISA kits (R&D Systems) according the manufacturer's instructions.

### Colony formation assay

The cells were seeded in a 6-well plate and cultured for 14 days. After washing twice with PBS, the cells were fixed with 4% paraformaldehyde (PFA) for 15 min and stained with crystal violet. Colonies with more than 50 cells were counted.

### Cell viability assay

Cell viability was determined using the Cell Counting Kit-8 (CCK-8) reagent (MCE). Briefly, the NPC cells were seeded in 96-well plates and cultured for 0-72h. At each time point, 10μL CCK-8 reagent was added to each well and the cells were incubated for 2h at 37°C. The absorbance was measured at 450 nm using a multi-mode plate reader (Molecular Devices).

### EdU assay

Edu assay was performed using the BeyoClick™ EdU Cell Proliferation Kit with Alexa Fluor 488 (Beyotime Biotech) according to the manufacturer's protocol. Briefly, the cells were incubated with EdU for 2h, fixed with 4% PFA for 15 min, and permeabilized with 0.3% Triton X-100 for 15 min. After incubating with the Click Reaction Mixture for 30 min in the dark, the cells were incubated with Hoechst 33342 for 10 min. Images were captured on a Leica TCS SP5 confocal microscope.

### Immunohistochemistry (IHC)

Tissue slides (Supplementary Table S3) were deparaffinized, covered with 3% hydrogen peroxide for 10 min to block endogenous peroxidase activity, and then immersed in Improved Citrate Antigen Retrieval Solution (Beyotime) at sub-boiling temperature for 10 min. After blocking in 10% goat serum (Beyotime) with 0.2% Triton X-100 (Beyotime) in PBS for 1h at room temperature, the slides were incubated with the primary antibody at 4°C in a humidified chamber overnight. Subsequently, the sections were incubated the secondary antibody and developed DAB color by using the GTVision III Detection System/Mo&Rb Kit (Gene Tech). Each specimen was scored according to the intensity of staining (0, none; 1, weak; 2, moderate; 3, strong) and the proportion of stained cells (0, 0%; 1, 1-24%; 2, 25-49%; 3, 50-74%; 4, 75-100%) according to the German semi-quantitative scoring system. The final immunoreactivity score was determined by multiplying the intensity with the positivity rate, and ranged from 0 to 12 15.

### Xenograft tumor model

All experiments were approved by the Animal Experimental Ethics Committee of Shenzhen Institute of Otolaryngology and Use of Laboratory Animals published by the US National Institute of Health. 4-week old male BALB/c nude mice (n=20) were purchased from Guangdong Medical Laboratory Animal Center. The tumor model was established by subcutaneously injecting nude mice with CNE1 or HONE1 cells expressing the SPZ1 shRNA or negative control. The volume (mm^3^) of the implanted tumor was calculated as (width^2^ × length)/2.

### Statistical analysis

Two-tailed Student's t-test was used to compare the data of two groups. Differences among three or more groups were compared by one-way ANOVA. Cell viability and tumor volume were analyzed by two-way ANOVA. All analyses were performed using the GraphPad Prism software version 5.0. Data were expressed as mean ± standard error of the mean (SEM) of at least three independent experiments. *P* value < 0.05 was considered statistically significant.

## Results

### SPZ1 is overexpressed in NPC samples

**Figure 1:**
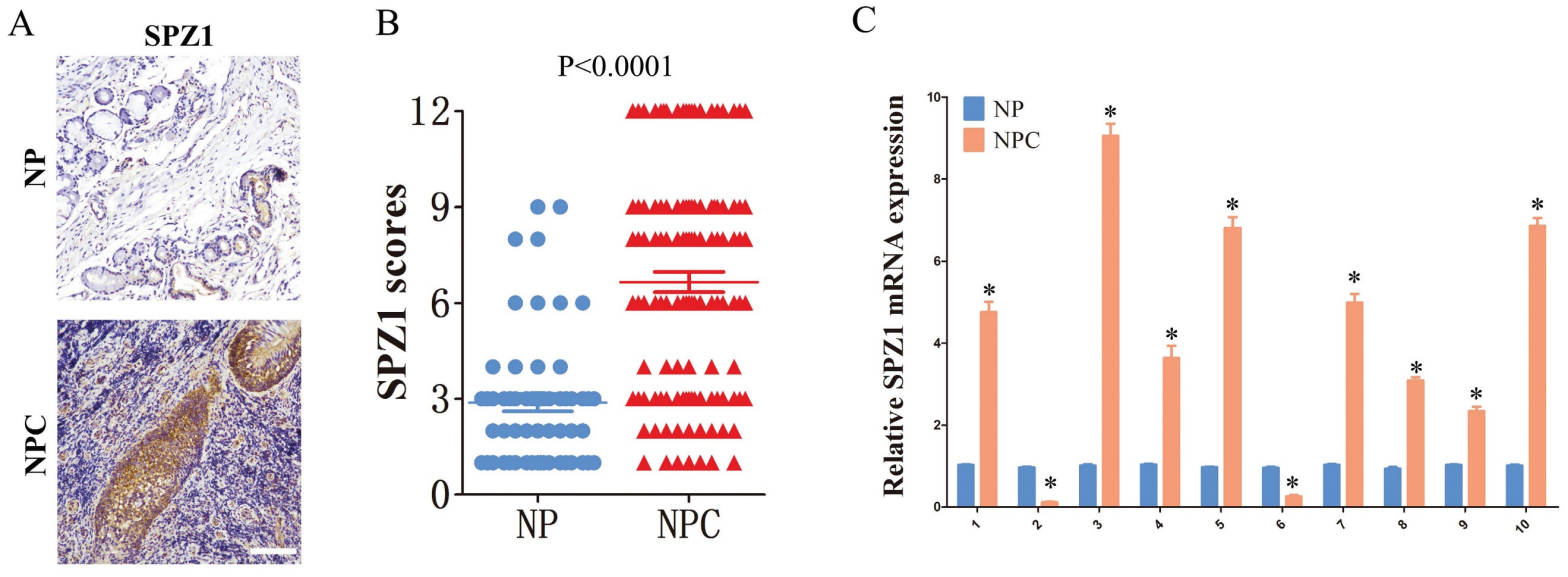
** SPZ1 expression in NPC tissues.** (A and B) Representative images and scores of SPZ1 protein expression in nasopharyngitis (NP) (n=58) and nasopharyngeal carcinoma (NPC) tissues (n=119). Scare bar = 100μm. (C) Relative SPZ1 mRNA levels in 10 paired tumor and normal nasopharyngeal tissues from NPC patients. * p<0.05.

The in-situ expression of SPZ1 protein in clinical samples was assessed by IHC. As shown in Figure 1A and B, the IHC scores of SPZ1 were significantly higher in the NPC samples compared to that in nasopharyngitis (NP) tissues. To further validate this finding, we compared the expression of SPZ1 mRNA between 10 paired human NPC tissues and adjacent normal tissues. As shown in Figure 1C, 8/10 NPC cases expressed higher SPZ1 mRNA compared to normal tissues. Taken together, these results indicate that SPZ1 is aberrantly upregulated in NPC.

### SPZ1 knockdown inhibited NPC cell growth *in vitro* and *in vivo*

**Figure 2:**
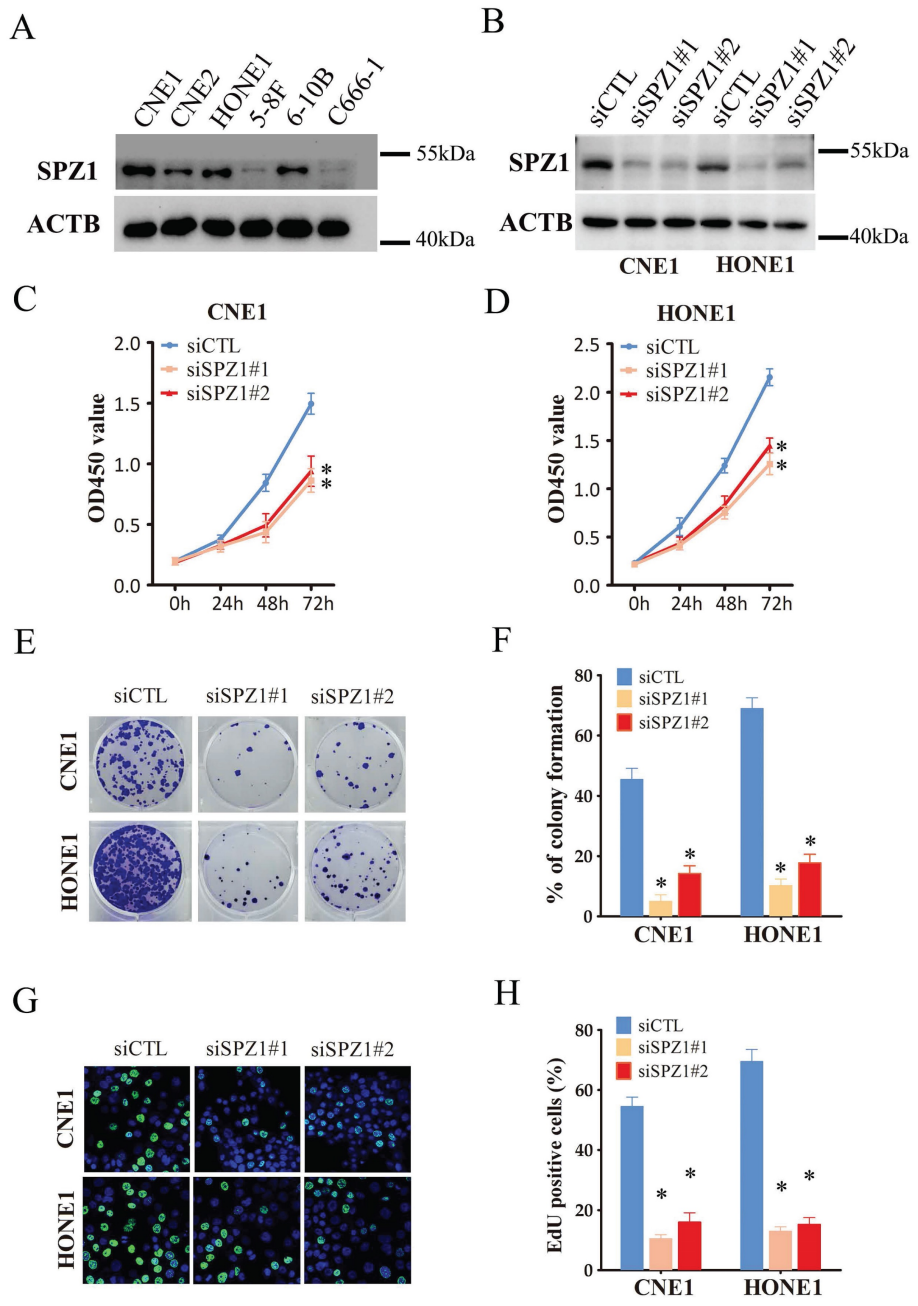
** SPZ1 knockdown inhibited NPC cells growth *in vitro*.** (A) SPZ1 protein expression in CNE1, CNE2, HONE1, 5-8F, 6-10B and C666-1 cell lines. (B) SPZ1 protein in CNE1 and HONE1 cells transfected with control siRNA (siCTL) or SPZ1 siRNA (siSPZ1#1 and siSPZ1#2). (C and D) Survival rates of CNE1 and HONE1 cells transfected with siCTL, siSPZ1#1 or siSPZ1#2. (E and F) Representative images and number of colonies formed by NPC cells transfected with siCTL, siSPZ1#1 or siSPZ1#2. (G and H) Representative images and number of Edu-positive proliferative in SPZ1 knockdown NPC cells. The data represent the mean ± SEM of at least three independent experiments. * p<0.05, versus siCTL.

**Figure 3:**
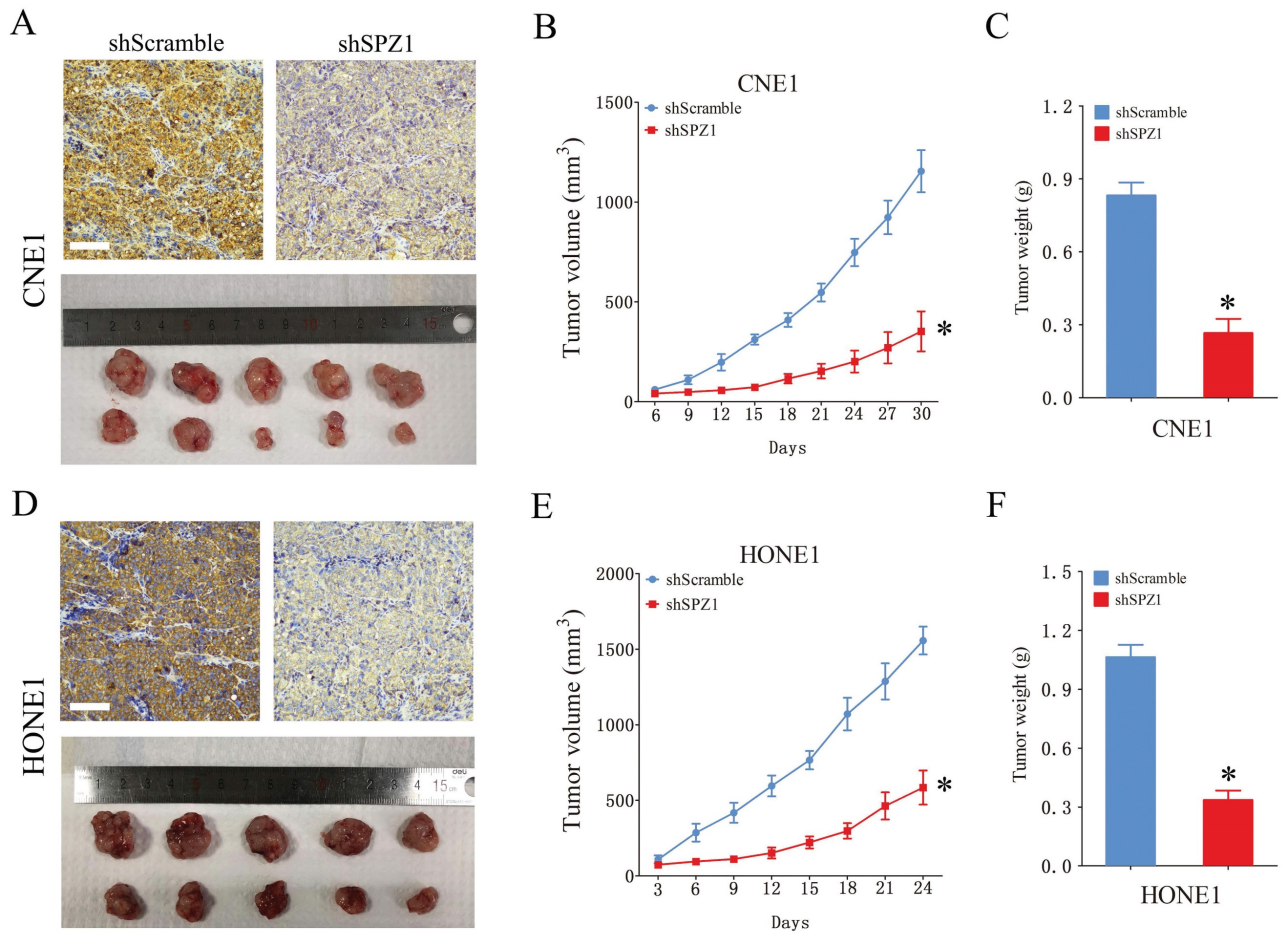
** SPZ1 knockdown inhibited NPC cells growth *in vivo*.** (A) Images of tumors and IHC showing SPZ1 expression in the xenografts of CNE1 cells transfected with shScramble or shSPZ1. (B and C) The tumor volume and weight in the xenografts of CNE1 cells. (D) Images of tumors and IHC showing SPZ1 expression in the xenografts of HONE1 cells transfected with shScramble or shSPZ1. (E and F) The tumor volume and weight in the xenografts of HONE1 cells. Scare bar = 100μm. The data represent the mean ± SEM, n=5. * p<0.05, versus shScramble.

We also analyzed the expression of SPZ1 protein in different human NPC cell lines, including CNE1, CNE2, HONE1, 5-8F, 6-10B and C666-1. SPZ1 levels were higher in the CNE1 and HONE1 cell lines (Figure 2A), which were selected for gene knockdown. As shown in Figure 2B, SPZ1 silencing significantly reduced SPZ1 protein expression in the CNE1 and HONE1 cells (siSPZ1#1 and siSPZ1#2). In addition, knocking down SPZ1 led to a significant decrease in the viability of NPC cells (Figure 2C and D), as well as in the number of colonies formed by both cell lines compared to their respective controls. Consistent with this, SPZ1 knockdown reduced the number of EdU-positive proliferative cells (Figure 2E-H). To establish a direct link between SPZ1 and the tumorigenic potential of NPC cells, we established subcutaneous tumors in nude mice with control (scrambled shRNA) or SPZ1-knockdown CNE1 or HONE1 cells. SPZ1 knockdown significantly decreased tumor volume and weight compared to that in the control group (Figure 3A-F). Taken together, SPZ1 is a key driver of NPC growth.

### SPZ1 knockdown suppressed the migration and invasion of NPC cells *in vitro*

**Figure 4:**
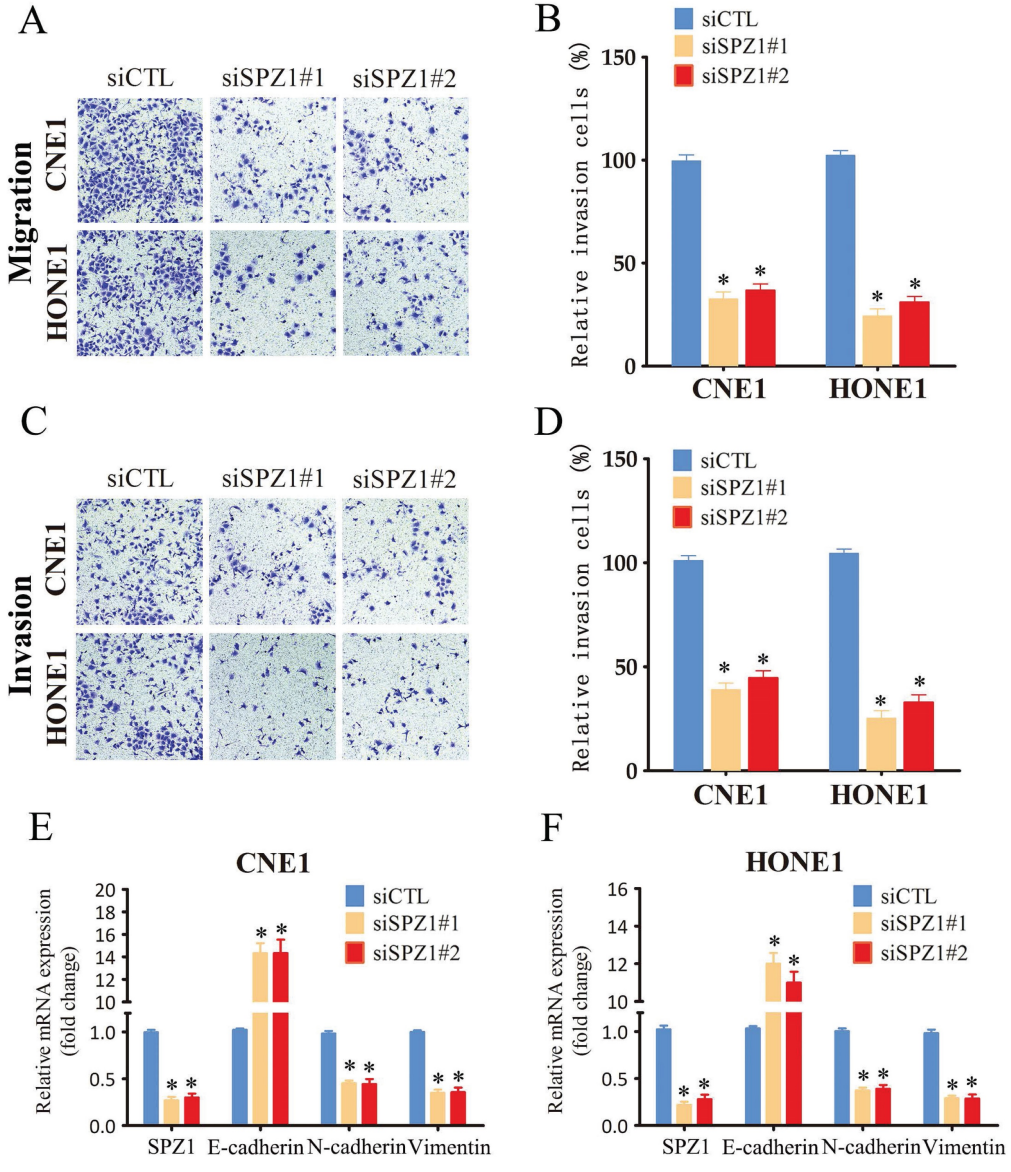
** SPZ1 knockdown inhibited NPC cells migration and invasion.** (A and B) Representative images and summary data of the migration assay in CNE1 and HONE1 cells transfected with siCTL, siSPZ1#1 or siSPZ1#2. (C and D) Representative images and summary data of the invasion assay in CNE1 and HONE1 cells transfected with siCTL, siSPZ1#1 or siSPZ1#2. (E and F) RNA levels of the epithelial-mesenchymal transition (EMT) markers in CNE1 and HONE1 cells transfected with siCTL, siSPZ1#1 or siSPZ1#2. The data represent the mean ± SEM of at least three independent experiments. * p<0.05, versus siCTL.

The impact of SPZ1 inhibition on the invasiveness of NPC cells was analyzed by the transwell assay. CNE1 and HONE1 cells transfected with siSPZ1 exhibited lower migration rates compared to the respective controls (Figure 4A and B). Likewise, SPZ1 knockdown also decreased the invasion ability of the NPC cells through Matrigel-coated transwell membrane compared to that of control cells (Figure 4C and D). EMT plays an important role in normal development as well as cancer metastasis via reversible induction of the mesenchymal phenotype 17. Therefore, we also analyzed the expression of EMT markers in the SPZ1-knockdown cells. SPZ1 silencing in the CNE1 and HONE1 cells significantly increased the expression of the epithelial marker E-cadherin, and reduced that of mesenchymal markers like N-cadherin and vimentin, which is indicative of EMT (Figure. 4E and F). Altogether, these findings strongly suggest that SPZ1 is critical for the metastasis and invasiveness of NPC cells.

### SPZ1 overexpression promoted the malignant potential of NPC cells *in vitro*

**Figure 5:**
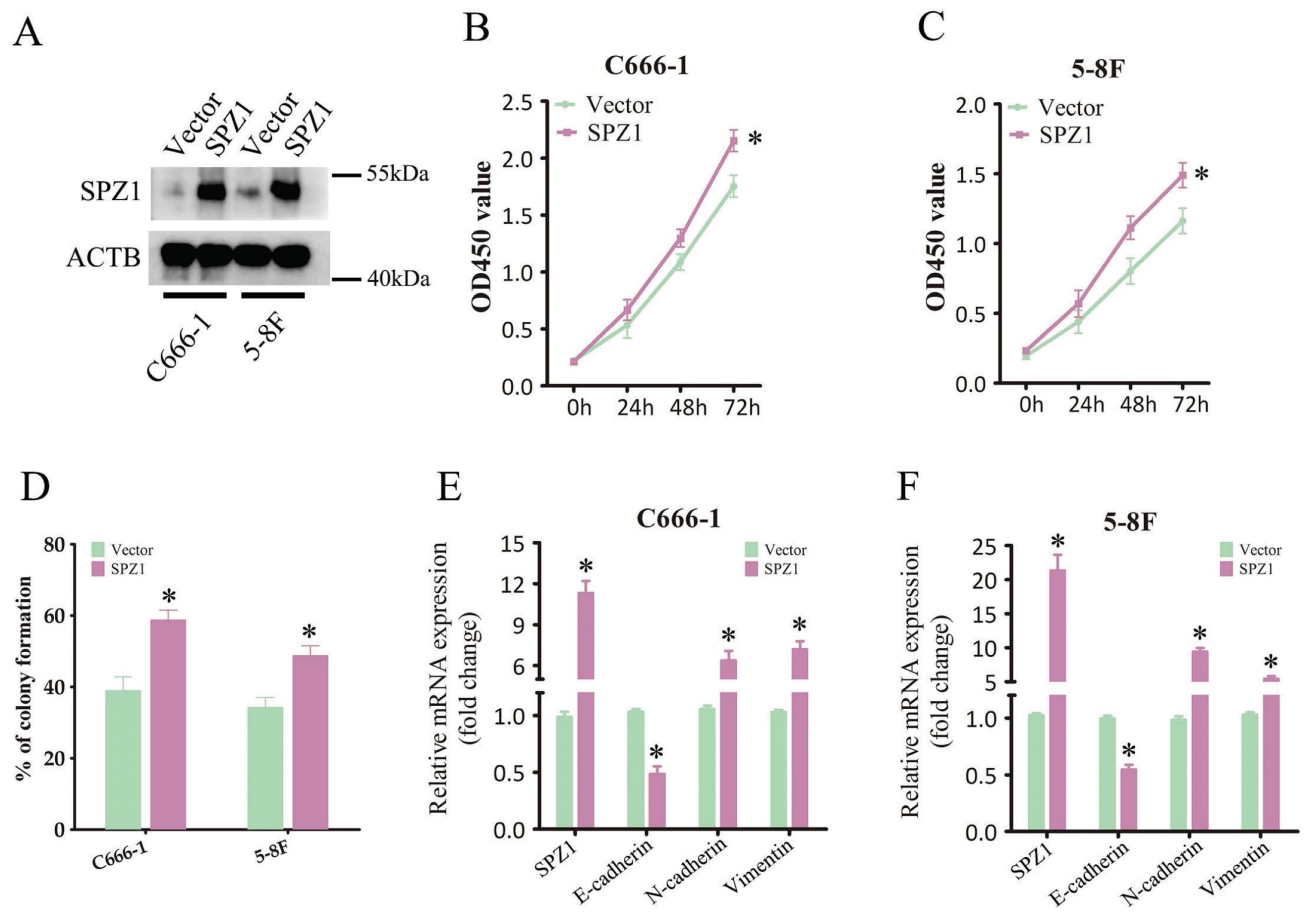
** Overexpression of SPZ1 promoted NPC cells progression.** (A) SPZ1 protein in C666-1 and 5-8F cells transfected with SPZ1-overexpression construct and vector. (B and C) Survival rates of C666-1 and 5-8F cells transfected with SPZ1-overexpression construct and vector. (D) Number of colonies formed by SPZ1 overexpression NPC cells. (E and F) RNA levels of SPZ1 and the epithelial-mesenchymal transition (EMT) markers in CNE1 and HONE1 cells C666-1 and 5-8F cells transfected with SPZ1-overexpression construct and vector. The data represent the mean ± SEM of at least three independent experiments. * p<0.05, versus vehicle.

To further explore the potential oncogenic mechanism of SPZ1 in NPC cells, we transfected C666-1 and 5-8F cells with full-length human SPZ1. As shown in Figure 5A, SPZ1 protein was upregulated in the transfected cells. Overexpression of SPZ1 significantly enhanced cell viability compared to that in the control group (Figure 5B and C), and increased the number of colonies formed *in vitro* (Figure 5D). In addition, SPZ1 overexpression also induced mRNA expression of EMT markers (Figure 5E and F). Altogether, these data suggest that SPZ1 functions as an oncogene in NPC.

### SPZ1-induced NPC progression depends on Wnt5a/IL-6 signaling

**Figure 6:**
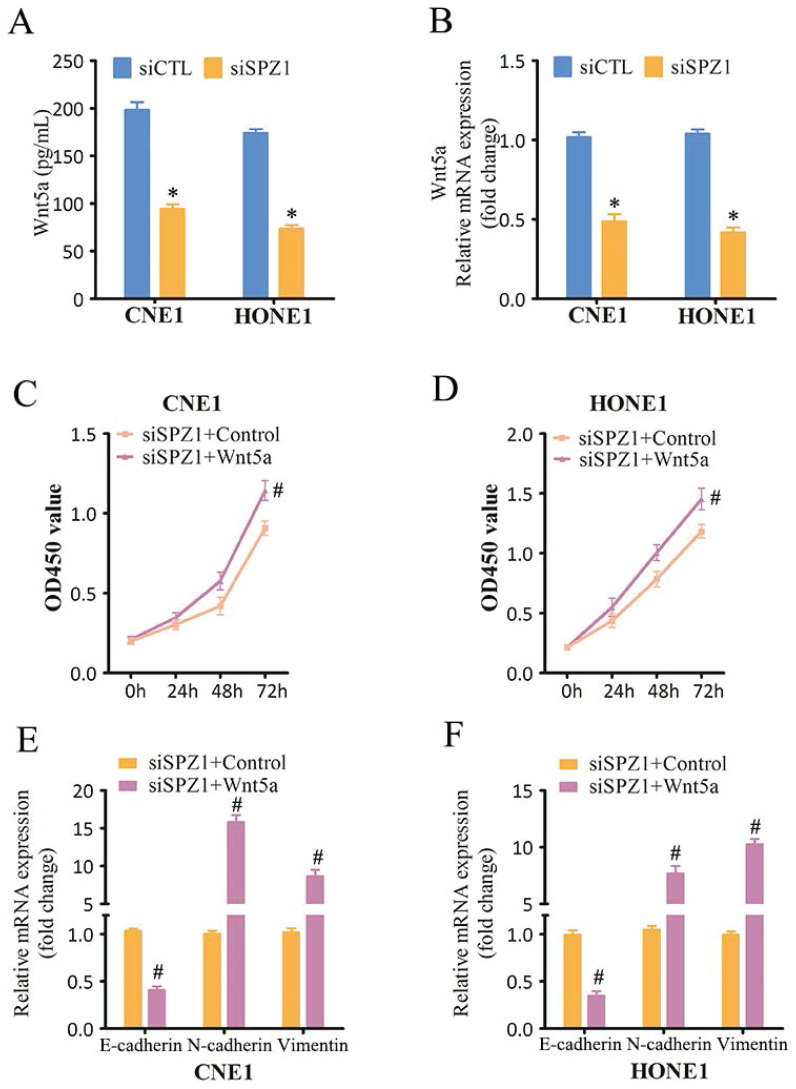
** SPZ1 knockdown inhibited Wnt5a expression in NPC cells.** (A) Wnt5a protein levels in the supernatants from CNE1 and HONE1 cells transfected with siCTL and siSPZ1. (B) Relative Wnt5a mRNA levels in SPZ1 konckdown NPC cells. (C and D) Survival rates of CNE1 and HONE1 transfected with siSPZ1 and pretreated with Wnt5a (300ng/mL). (E and F) RNA levels of the epithelial-mesenchymal transition (EMT) markers in CNE1 and HONE1 cells transfected with siSPZ1 and pretreated with Wnt5a. The data represent the mean ± SEM of at least three independent experiments. * p<0.05, versus siCTL or siCTL+Control.

Wnt5a belongs to the Wnt family of cysteine-rich secreted glycoproteins that are involved in NPC progression 16. SPZ1 promotes liver oncogenesis by transcriptionally activating Wnt5a 11. Consistent with this, SPZ1 silencing in the CNE1 and HONE1 cells decreased the expression of Wnt5a (Figure 6A and B). To further verify whether SPZ1 drives NPC progression in a Wnt5a-dependent manner, the SPZ1-knockdown NPC cells were pre-treated with Wnt5a. As shown in Figure 6C and D, Wnt5a stimulation reversed the inhibitory effects of SPZ1 knockdown in the NPC cells. Consistent with these findings, exogenous Wnt5a also downregulated E-cadherin, and upregulated N-cadherin and Vimentin in the SPZ1-knockdown cells (Figure 6E and F). Taken together, these data indicated that Wnt5a mediates the oncogenic effects of SPZ1 in NPC progression.

**Figure 7:**
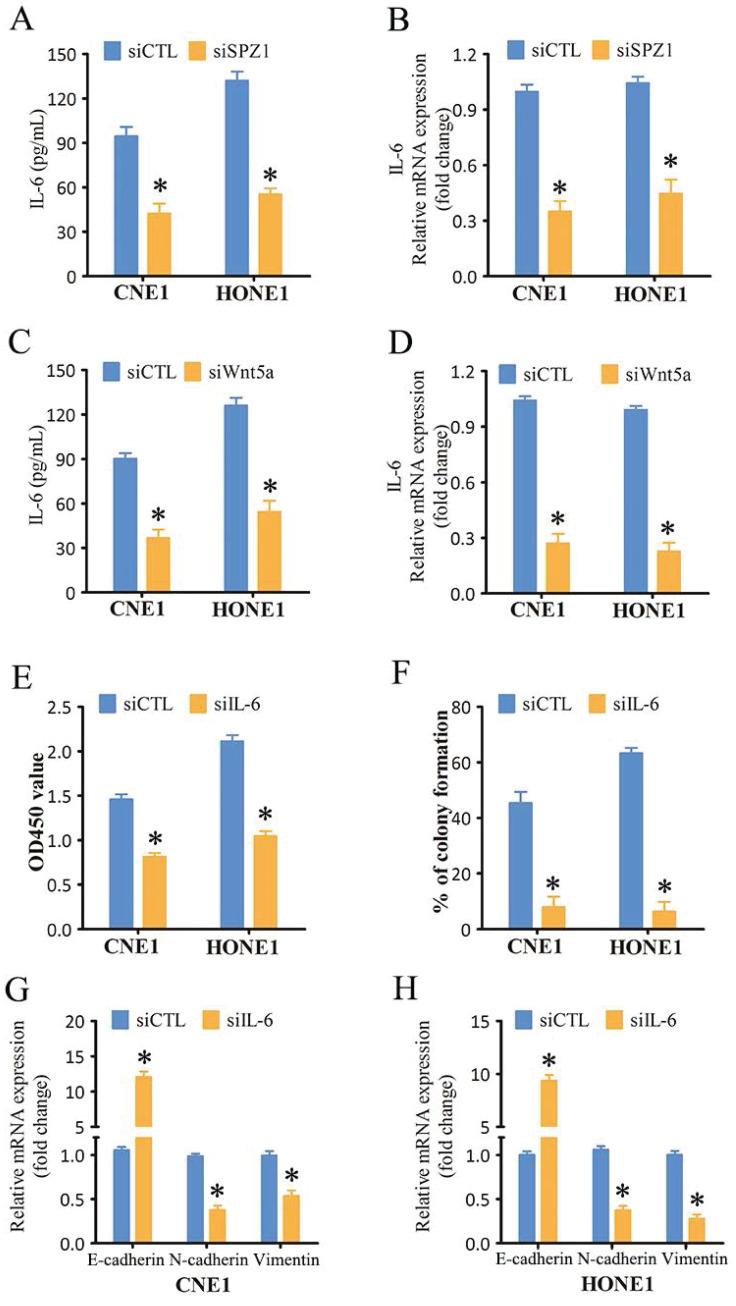
** Inhibition of SPZ1/Wnt5a suppressed IL-6 expression in NPC cells.** (A) IL-6 protein levels in the supernatants from CNE1 and HONE1 cells transfected with siCTL and siSPZ1. (B) Relative IL-6 mRNA levels in SPZ1 konckdown NPC cells. (C) IL-6 protein levels in the supernatants from CNE1 and HONE1 cells transfected with siCTL and siWnt5a. (D) Relative IL-6 mRNA levels in Wnt5a konckdown NPC cells. (E and F) Survival rates and number of colonies formed by NPC cells transfected with siIL-6. (G and H) RNA levels of the epithelial-mesenchymal transition (EMT) markers in NPC cells transfected with siIL-6. The data represent the mean ± SEM of at least three independent experiments. * p<0.05, versus siCTL.

IL-6 plays a key role in the proliferation and metastasis of various tumor cells 17. SPZ1 silencing in the CNE1 and HONE1 cells decreased the levels of secreted IL-6 compared to that in the control group (Figure 7A). Similar trends were also observed for IL-6 mRNA expression (Figure 7B). In addition, IL-6 levels were also decreased in the xenograft tumors derived from SPZ1-knockdown cells (Figure S3). To further verify whether SPZ1-regulates IL-6 expression in a Wnt5a-dependent manner, we knocked down Wnt5a in the NPC cells. As shown in Figure 7C and D, Wnt5a silencing attenuated IL-6 expression. Finally, IL-6 knockdown in the NPC cells markedly decreased their proliferation (Figure 7E and F) and EMT (Figure 7G and H). Taken together, the oncogenic effects of SPZ1 are mediated via the Wnt5a/IL-6 signaling axis.

### IL-6 mediates the oncogenic effects of SPZ1 in NPC

**Figure 8:**
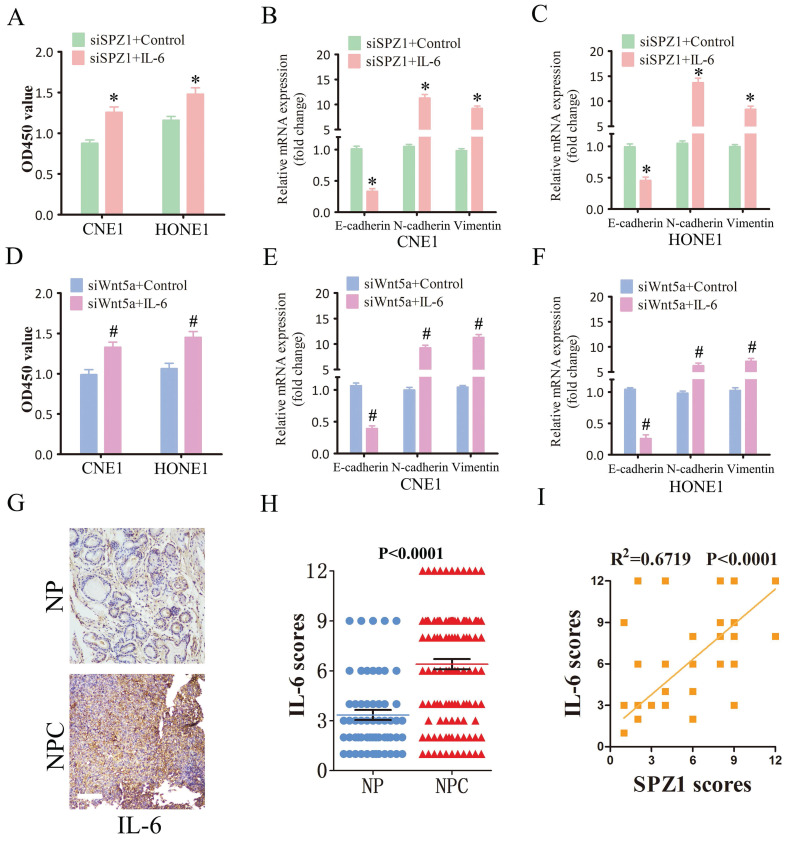
** SPZ1-mediated Wnt5a promoted NPC progression through IL-6.** (A) Survival rates of CNE1 and HONE1 transfected with siSPZ1 and pretreated with IL-6 (100ng/mL). (B and C) RNA levels of the epithelial-mesenchymal transition (EMT) markers in CNE1 and HONE1 cells transfected with siSPZ1 and pretreated with IL-6 (100ng/mL). (D) Survival rates of CNE1 and HONE1 transfected with siWnt5a and pretreated with IL-6 (100ng/mL). (E and F) RNA levels of the EMT markers in CNE1 and HONE1 cells transfected with siWnt5a and pretreated with IL-6 (100ng/mL). (G and H) Representative images and scores of IL-6 protein expression in nasopharyngitis (NP) (n=58) and nasopharyngeal carcinoma (NPC) tissues (n=119). (F) Pearson correlation of IL-6 and SPZ1 expression levels. Scare bar = 100μm.The data represent the mean ± SEM of at least three independent experiments. * p<0.05, versus siSPZ1+Control, siWnt5a+Control or NP.

To further verify whether SPZ1-mediated progression of NPC depends on IL-6, we treated SPZ1-knockdown NPC cells with the IL-6. As shown in Figure 8A, IL-6 reversed the inhibitory effects of SPZ1 knockdown, and restored EMT of the NPC cells (Fig. 8B and C). Furthermore, IL-6 also overcame the repressive effect of Wnt5a knockdown on the growth of NPC cells (Fig. 8D to F). Consistent with the *in vitro* results, IL-6 protein expression was higher in the NPC tissues compared to that in the NP tissues (Fig. 8G and H). In addition, IL-6 expression in the tumor tissues correlated positively with SPZ1 expression (R^2^ =0.6719; Figure 8I).

## Discussion

NPC is one of the most misdiagnosed diseases, and has a high incidence rate, particularly in southern China, Southeast Asia, North Africa, and the Arctic^18^,^19^. Radiotherapy and chemotherapy are the primary options for the clinical management of NPC, although these treatment regimens often reduce the quality of life of the patients. Therefore, it is crucial to understand the molecular mechanisms underlying NPC tumorigenesis, and identify effective diagnostic biomarkers and new therapeutic targets for improving survival rates and prognoses. There is growing evidence that cancer progression is associated with aberrant SPZ1 expression in tumor tissues 11,13,14,20. The role of SPZ1 in the progression of NPC remains uncertain. We have shown for the first time that SPZ1 promotes NPC progression by increasing IL-6 production via Wnt5a signaling.

Liu *et al.* and Wang *et al.* showed that SPZ1 promotes EMT of breast and liver tumor cells respectively in a Twist1-dependent manner 11,13,14. Furthermore, SPZ1 inhibits apoptosis of colorectal cancer cells, promotes proliferation, and enhances resistance to 5-fluorouracil 11,13,14. These findings have been confirmed in *in vitro* and *in vivo* models, indicating an oncogenic function of SPZ1. In this study, we selected the CNE1 and HONE1 cell lines with high SPZ1 expression, and successfully knocked down the SPZ1 gene. Knocking down SPZ1 inhibited the proliferation and invasiveness of NPC cells *in vitro*, and impaired the growth of xenografts *in vivo*. Overexpression of SPZ1 enhanced malignant potential of C666-1 and 5-8F cells. These findings are consistent with previous results, and indicate that SPZ1 plays a key role in NPC progression.

WNT5a is an oncogene that promotes the EMT and metastasis of tumor cells. Overexpression of WNT5a increased colony formation, migration, and invasion of NSCLC cells and induced EMT, while WNT5a knockout had the opposite effect 21. Furthermore, Zhu *et al.* had shown that Wnt5a regulates the EMT of NPC cells, and affects tumor invasion and metastasis 22. Therefore, we hypothesized that SPZ1 may promote NPC progression in a Wnt5a-dependent manner. SPZ1 silencing in the CNE1 and HONE1 cells reduced Wnt5a expression, while exogenous Wnt5a alleviated the inhibitory cell growth of SPZ1 knockdown. Furthermore, Wnt5a also restored the EMT of SPZ1-knockdown cells by downregulating E-cadherin, and upregulating N-cadherin and vimentin. E-cadherin is a calcium-dependent cell adhesion molecule that maintains cellular morphology and structural integrity by mediating homotypic cell-cell adhesion. Reduction in E-cadherin levels leads to the loss of cell-cell adhesion, basement membrane disruption, and decreased epithelial or endothelial stability, eventually resulting in metastasis. High expression levels of N-cadherin and vimentin are associated with enhanced tumor metastasis and invasion. Upregulation of N-cadherin is linked to tumor growth, migration, invasion, lymph node metastasis and EMT. On the other hand, high vimentin expression alters cytoskeletal protein structures, which promotes the transformation of cuboidal epithelial cells into spindle-shaped fibroblast-like cells, and enhances their migratory capacity 23,24. Exogenous Wnt5a partially restored the proliferation and invasion of SPZ1-knockdown NPC cells, further validating the role of Wnt5a in SPZ1-induced NPC progression.

Various cytokines are involved in tumor formation, progression and metastasis, and form a functional link between inflammation and cancer 25-27. Several studies have reported high serum levels of IL-6 in NPC patients, which also correlates with advanced stages of the disease 28. In addition, high IL-6 expression in NPC tissues contributes to disease progression and poor outcomes 29. Based on these findings, we hypothesized that IL-6 may mediate the oncogenic effects of SPZ1. Indeed, SPZ1 knockdown in CNE1 and HONE1 cells reduced IL-6 expression at both mRNA and protein levels. Furthermore, Wnt5a silencing in these cells also downregulated IL-6. Previous studies have shown that melanoma cells with Wnt5a knockdown secrete lower levels of IL-6 compared to control cells 30, and activation of Wnt5a signaling in these cells enhanced IL-6 expression 31. These results suggest that SPZ1 regulates IL-6 expression via the Wnt5a pathway. IL-6 is a critical factor in promoting tumor development 32,33. IL-6 silencing in NPC cells significantly impaired cell growth and proliferation, while exogenous IL-6 restored the malignant features of the SPZ1-knockdown NPC cells, indicating that IL-6 participates in SPZ1-mediated tumor progression. Similarly, IL-6 also neutralized the inhibitory effects of Wnt5a silencing, suggesting its involvement in Wnt5a-induced oncogenesis. Furthermore, IL-6 expression was higher in the NPC tissues compared to NP tissues, and correlated positively with SPZ1 expression, further indicating that IL-6 is involved in NPC progression. However, the transcriptional or translational mechanism of IL-6 should be investigated in further study.

## Conclusion

In conclusion, our findings reveal a novel mechanism through which SPZ1 mediates the growth and invasion of NPC. Briefly, SPZ1 activates the Wnt5a/IL-6 signaling cascade, which contributes to NPC progression. Therefore, the SPZ1/ Wnt5a/IL-6 axis is a potential therapeutic target for NPC.

## Supplementary Material

Supplementary figures and tables.
