# Supplementary material for: Induction of interleukin-6 by SPZ1-mediated Wnt5a signaling boosts progression of nasopharyngeal carcinoma cells
Source: J Cancer. 2024 Oct 7;15(18):6148–59. doi: 10.7150/jca.99648 (PMC11493014; doi:10.7150/jca.99648)
Supplement: Supplementary file 1 — Supplementary figures and tables. [file jcav15p6148s1.pdf]

Supplementary information

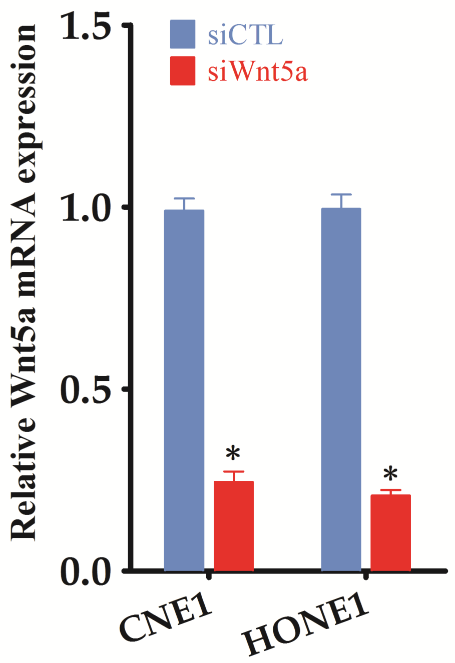

Figure S1. Wnt5a mRNA levels in CNE1 and HONE1 cells transfected with siRNA against Wnt5a. Values are means ± SEM, n=4 *, p<0.05 compared to siCTL.

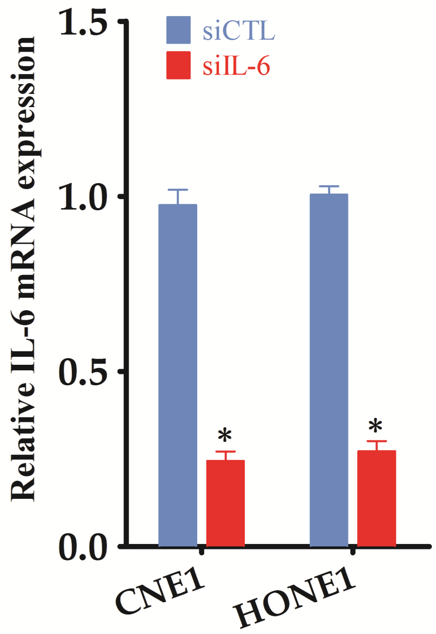

Figure S2. IL-6 mRNA levels in CNE1 and HONE1 cells transfected with siRNA against IL-6. Values are means ± SEM, n=4 *, p<0.05 compared to siCTL.

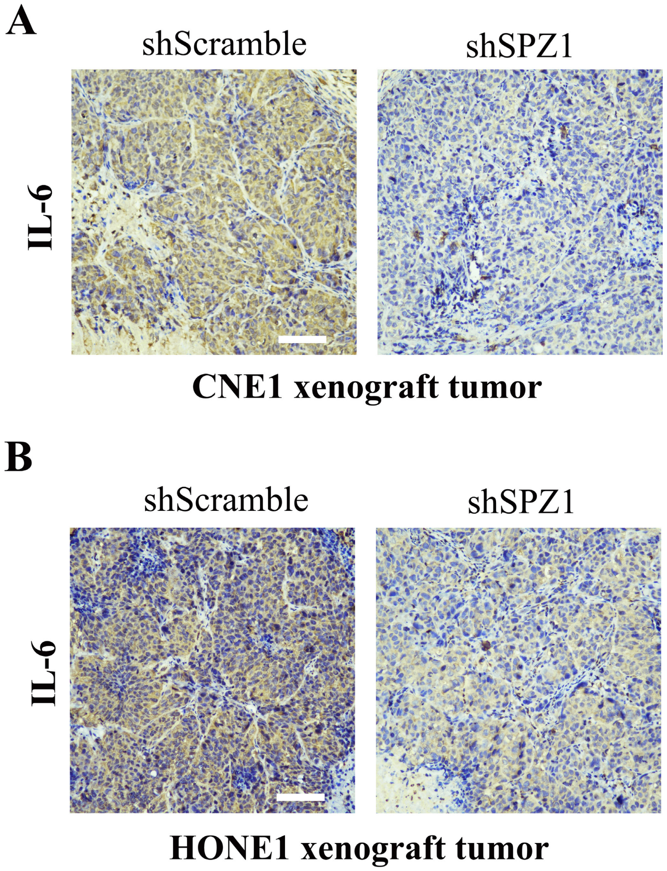

Figure S3. (A and B) Images of IHC showing IL-6 expression in the xenografts of CNE1 or HONE1 cells transfected with shScramble or shSPZ1. Scare bar = 100μm.

Table S1 Primer sequences used for Real-time PCR

Gene Name	Primer	Sequence
SPZ1	Forward<br>Reverse	GGAACAGGTG AAGAAACTGAGCC<br>GCTTCTCTTGCAGAGTTCCCTG
E-cadherin	Forward<br>Reverse	GCCTCCTGAAAAGAGAGTGGAAG<br>TGGCAGTGTCTCTCCAAATCCG
N-cadherin	Forward<br>Reverse	CCTCCAGAGTTTACTGCCATGAC<br>GTAGGATCTCCGCCACTGATTC
Vimentin	Forward<br>Reverse	AGGCAAAGCAGGAGTCCACTGA<br>ATCTGGCGTTCCAGGGACTCAT
Wnt5a	Forward<br>Reverse	TACGAGAGTGCTCGCATCCTCA<br>TGTCTTCAGGCTACATGAGCCG
IL-6	Forward<br>Reverse	AGACAGCCACTCACCTCTTCAG<br>TTCTGCCAGTGCCTCTTTGCTG
Actin	Forward<br>Reverse	CACCATTGGCAATGAGCGGTTC<br>AGGTCTTTGCGGATGTCCACGT

Table S2 Target sequences used for silencing

Gene Name	Target sequence
SPZ1	siRNA#1: 5’-CCATTGCCTTATTCGAAAT-3’<br>siRNA#2 :5’-CCATCAAGTTACAGAACAA-3’
Wnt5a	siRNA:5’- GCCAGUAUCAAUUCCGACA-3’
IL-6	siRNA: 5’-CUUCCAAUCUGGAUUCAAU-3’

Table S3 Clinicpathological characteristics of NPC patients

Characteristic	All Patients (n=119) n	All Patients (n=119) %
Age(years)		
≤50	39	32.8
>50	80	67.2
Sex		
Male	96	80.7
Female	23	19.3
T stage		
T1-T2	48	40.3
T3-T4	71	59.7
N stage		
N0-1	76	63.9
N2-3	43	36.1
M stage		
M0	57	47.9
M1	62	52.1
TNM stage		
I-II	73	61.3
III-IV	46	38.7
Smoking		
Yes	87	73.1
No	32	26.9
